# Confirmed Angiosarcoma: Prognostic Factors and Outcome in 50 Prospectively Followed Patients

**DOI:** 10.1155/2000/575781

**Published:** 2000-12-22

**Authors:** N. J. Espat, J. J. Lewis, J. M. Woodruff, C. Antonescu, J. Xia, D. Leung, Brennan M. F.

**Affiliations:** ^1^ Department of Surgery, Memorial Sloan-Kettering Cancer Center, New York, New York, USA; ^2^ Department of Pathology, Memorial Sloan-Kettering Cancer Center, New York, New York, USA; ^3^ Department of Biostatistics, Memorial Sloan-Kettering Cancer Center, New York, New York, USA; ^4^ University of Illinois at Chicago, Department of Surgery M/C 958, 840 South Wood Street, Room 435E, Chicago, IL 60612, USA

## Abstract

*Purpose*. Angiosarcoma is a rare tumor with endothelial cell differentiation that may arise in any anatomic location.The purpose of this report was to identify prognostic factors on outcome in a group of prospectively followed patients with confirmed angiosarcoma.

*Subjects*. Adult patients (>16 years old) with angiosarcoma treated between July 1982 and February 1998 were identified from a prospective database.

*Methods*. Pathologic confirmation of all cases was performed prior to inclusion in this analysis. Various prognostic factors were evaluated for disease-specific survival. Survival was determined by the Kaplan– Meier method. Statistical significance was evaluated by log-rank test for univariate analysis and Cox stepwise regression for multivariate analysis (p < 0.05).

*Results*. Fifty patients were identified; at the initial evaluation, this group included 32 patients with a primary tumor, three with local recurrence and 15 with metastatic disease. Tumor sites included 16 head and neck and skin of head, eight extremity, seven trunk, six breast, five pelvis, four viscera and four thoracic. Median follow-up among survivors was 71 months (range, 38–191 months).Two- and 5-year disease-specific survival was 50 and 30%, respectively, with a median of 24 months. The factor predictive of tumor-related mortality was presentation status (p=0.001; relative risk, 5). Two-year disease-specific survival for patients presenting with recurrent or metastatic disease was 13%, compared with 70% for those with primary disease.


*Sarcoma* (2000) **4**, 173–177

ORIGINAL ARTICLE

### Confirmed angiosarcoma: prognostic factors and outcome in 50 prospectively followed patients

N. J. ESPAT,[1] J. J. LEWIS,[1] J. M. WOODRUFF,[2] C. ANTONESCU,[2] J. XIA,[2] D. LEUNG[3] & M. F. BRENNAN[1]

[1]*Department of Surgery,* [2]*Department of Pathology and* [3]*Department of Biostatistics, Memorial Sloan-Kettering Cancer Center, New York, New York, USA*

**Abstract**

*Purpose.* Angiosarcoma is a rare tumor with endothelial cell differentiation that may arise in any anatomic location. The purpose of this report was to identify prognostic factors on outcome in a group of prospectively followed patients with confirmed angiosarcoma.

*Subjects.* Adult patients (>16 years old) with angiosarcoma treated between July 1982 and February 1998 were identified from a prospective database.

*Methods.* Pathologic confirmation of all cases was performed prior to inclusion in this analysis. Various prognostic factors were evaluated for disease-specific survival. Survival was determined by the Kaplan–Meier method. Statistical significance was evaluated by log-rank test for univariate analysis and Cox stepwise regression for multivariate analysis ($p<0.05$).

*Results.* Fifty patients were identified; at the initial evaluation, this group included 32 patients with a primary tumor, three with local recurrence and 15 with metastatic disease. Tumor sites included 16 head and neck and skin of head, eight extremity, seven trunk, six breast, five pelvis, four viscera and four thoracic. Median follow-up among survivors was 71 months (range, 38–191 months). Two- and 5-year disease-specific survival was 50 and 30%, respectively, with a median of 24 months. The factor predictive of tumor-related mortality was presentation status ($p=0.001$; relative risk, 5). Two-year disease-specific survival for patients presenting with recurrent or metastatic disease was 13%, compared with 70% for those with primary disease.

*Discussion.* Angiosarcoma is an aggressive sarcoma with high metastatic potential and subsequent mortality. Patients with primary angiosarcoma demonstrate a survival advantage compared with patients with metastatic disease. Enrollment in investigational trials for high-risk patients with recurrent or metastatic disease is recommended to potentially improve survival.

### Introduction

Vascular sarcomas are uncommon vasoformative soft tissue tumors. Historically, angiosarcoma, lymphangiosarcoma and hemangiopericytoma have been grouped under the heading of vascular soft tissue sarcomas.[1] Hemangiopericytoma is thought to arise from specialized smooth muscle cells termed 'Pericytes of Zimmerman' that surround blood vessels. In contrast, angiosarcoma and lymphangiosarcoma demonstrate endothelial cell differentiation. Pathologically, there is no difference between angiosarcoma and lymphangiosarcoma, a tumor whose distinction is based on its clinical presentation, and both are included in this study. Epithelioid hemangioendothelioma[2] is a more recently described malignant vascular tumor that, from preliminary statements,[3] seems to follow a less malignant course than angiosarcoma and is not included in the present analysis.

Angiosarcoma represents about 2% of all soft tissue sarcomas.[4] At the time of diagnosis, 10–25% of patients already have metastatic disease.[4] Angiosarcoma may originate from any anatomic site, although the most common sites are the skin of the head and neck, extremities, trunk and breast;[4,5] but angiosarcoma of the liver, lungs, heart, pelvis and retroperitoneum has also been reported. The development of angiosarcoma in patients undergoing prior irradiation,[6] toxic chemical exposure, Stuart–Treves syndrome and other malignancies[4,7] has been documented but, for the majority of patients, no specific risk factors are identified. Similarly, limited information is available on prognostic factors that affect outcome for patients with angiosarcoma.

### Methods

A prospective database of adult patients (older than 16 years) with soft tissue sarcoma, admitted and

Correspondence to: N. Joseph Espat, MD, Assistant Professor of Surgery, University of Illinois at Chicago, Department of Surgery M/C 958, 840 South Wood Street, Room 435E, Chicago, IL 60612, USA. Tel. +1 312 355-1493; Fax. +1 312 355-1987; E-mail: jespat@uic.edu

1357-714X print/1369-1643 online/00/040173-05 © 2000 Taylor & Francis Ltd

*DOI: 10.1080/13577140020025896*

treated at the Memorial Sloan–Kettering Cancer Center, was established in July 1982. The database contained 73 patients who underwent treatment for angiosarcoma including lymphangiosarcoma between July 1982 and February 1998.

Inclusion into this analysis was restricted to patients with a confirmed diagnosis of angiosarcoma or lymphangiosarcoma; pathologic assessment was conducted by three experienced soft-tissue sarcoma pathologists (J.M.W., C.A., J.X.). Only those cases with a unanimous agreement as to the diagnosis were included in this analysis. In 15/73 cases, pathological material was not available or was insufficient for evaluation. Five out of 73 patients had tumors that were considered undifferentiated sarcomas, 1/73 had an epithelioid hemangioendothelioma, and 2/73 patients had benign vascular tumors. The remaining 50 cases form the basis of this report.

Clinical and pathologic variables were correlated with survival endpoints. Various chemotherapy and radiation therapy regimens were used to treat these patients. However, over the 16-year period, differences in chemotherapy agents available, radiation protocols, patient enrollment into clinical trials and the geographic availability of these modalities precludes their analysis as a predictive factor. Clinical variables analyzed included: age at diagnosis (<50 or >50 years) and sex. Tumor variables analyzed included: microscopic margins (negative or positive), size (<5, 5–10 and >10 cm), depth (superficial or deep), and anatomic site (head and neck and skin of head, extremity, trunk, breast, pelvis/retroperitoneum, viscera and thoracic cavity). Tumors were considered high grade with the exception of angiosarcomas of the breast, which were graded according to the criteria of Donnell *et al.*[8] using a three-tier grading scheme; however, histologic grade was not a variable in this study as there were insufficient numbers of patients with low-grade lesions for analysis.

### Survival and statistical analysis

Disease-specific survival was calculated by the method of Kaplan and Meier.[9] Deaths caused by the disease were treated as an endpoint for disease-specific survival. Other deaths were treated as censored observations.

Significance between survival curves of populations was evaluated using log-rank testing for univariate influence and Cox-model stepwise regression for multivariate influence. The results of the Cox model analysis are reported with relative risks and confidence intervals. Comparison between clinical and pathological categorical variables in different groups was performed using Fisher's exact test for univariate and multivariate logistic regression to establish independent adverse prognostic factors for disease-specific survival. In all statistical analyses, $p<0.05$ was considered significant.

### Results

### *Patients*

Fifty patients (25 male and 25 female) were identified. Their ages ranged from 18 to 84 years with a median of 55 years. Fifty-eight percent of the patients were over 50 years old. At presentation, 32 patients (64%) had primary tumor, three (6%) had a local recurrence and 15 (30%) had metastatic disease (Table 1). One patient in each of the primary and metastatic tumor presentation categories had clinical evidence of lymphangiosarcoma. Head, neck and skin of head (HNS) was the most common anatomic site of disease in 16 patients (32%), followed in frequency by the extremities in eight (16%), trunk in seven (14%), breast in six (12%), pelvis/retroperitoneum in five (10%), viscera in four (8%) and thorax in four patients (8%).

Patients presenting with recurrence or metastasis had a higher proportion of tumors >5 cm than those with localized disease (12/18 versus 9/32; $p$=0.001). There were 14/18 deep tumors in the former group versus 14/32 in the latter ($p$=0.02).

### *Pathologic findings*

With the exception of four mammary tumors, all angiosarcomas were high grade. The mammary tumors showed a range of low- to high-grade angiosarcoma.

Surgical resection was performed in 29/32 of the patients presenting with primary tumor. Following resection, six patients (20%) had positive gross margins and 12 (41%) had positive microscopic margins.

**Table 1.** *Clinical characteristics of 50 patients with angiosarcoma*

| | *n* | % of total |
|---|---|---|
| Primary | 32 | 64 |
| Local recurrence | 3 | 6 |
| Metastatic | 15 | 30 |
| Gender | 25 M, 25 F | |
| Age range (years) | 18–84 | |
| Median age (years) | 55 | |
| Size | | |
| Unknown | 3 | 6 |
| <5 cm | 29 | 58 |
| ≥5 and <10 cm | 13 | 26 |
| ≥10 cm | 5 | 10 |
| Depth | | |
| Superficial | 22 | 44 |
| Deep | 28 | 56 |
| Site | | |
| Head, neck and scalp | 16 | 32 |
| Extremities | 8 | 16 |
| Trunk | 7 | 14 |
| Breast | 6 | 12 |
| Pelvis/retroperitoneum | 5 | 10 |
| Viscera | 4 | 8 |
| Thoracic | 4 | 8 |

*Follow-up*

Median follow-up for survivors was 71 months (range, 38–191 months). During the follow-up period, eight (16%) patients developed local recurrence and 14 (28%) metastases.

Of the initial 50 patients, 33 (66%) patients died of disease and seven (14%) patients died of other causes during the follow-up period. When grouped by presentation status, 15 (44%) patients presenting with primary disease, three (100%) patients presenting with a local recurrence and 15 (100%) patients presenting with metastatic disease succumbed to their disease.

*Survival*

The 2- and 5-year overall survival was 50 and 30%, respectively (Fig. 1), with a median overall survival of 24 months. The 2-year disease-specific survival for those presenting with locally recurrent disease was 33%, and was 13% for those presenting with metastases, compared with 70% for patients presenting with primary disease (Fig. 2). All patients with recurrent or metastatic angiosarcoma died, and they had five times the risk of dying from disease than the primary presenters ($p$=0.001). No other covariates were found to be independent adverse prognostic factors for disease-specific survival.

### Discussion

Angiosarcoma of the HNS was the most frequent anatomic site of disease in this analysis; an observation that is consistent with Western and Japanese literature.[4,10] HNS angiosarcoma occurred in approximately one-third of the entire patient group ($n$=50), as well as in 41% of the patients in the primary-presentation group ($n$=32).

Patients that presented with recurrent or metastatic disease demonstrated a significant decrease in overall survival. Metastatic disease at presentation has been a consistent factor associated with a worsened survival for patients with angiosarcoma[5] and for patients with other sarcoma subtypes.[11–13] In this cohort, the 2-year disease-specific survival for patients presenting with recurrent or metastatic disease was 13%, compared with 70% for those with primary disease.

Of the patients with primary disease at presentation who underwent surgical resection ($n$=29), six (20%) had positive gross margins and 12 (41%) had positive microscopic margins following resection. These data suggest that resection of gross disease may not achieve microscopic negative margins, since positive microscopic margins were identified twice as often as positive gross margins. This may be due to biologic factors such as unappreciated tumor extension or multicentric disease.[5,14] Furthermore, the inability to achieve microscopically negative margins for patients with angiosarcoma has been consistently identified and has been associated with an unfavorable survival outcome.[13–15] Because of the modest size of our cohort, we could not confirm this observation.

Tumor extension beyond the primary resection site and the need for subsequent re-resection for patients with soft tissue sarcoma (STS) is well described.[15] Patients with STS that have undergone a prior resection and obtained negative microscopic margins, upon undergoing re-resection as an oncologic principle, may be found to have residual disease in the

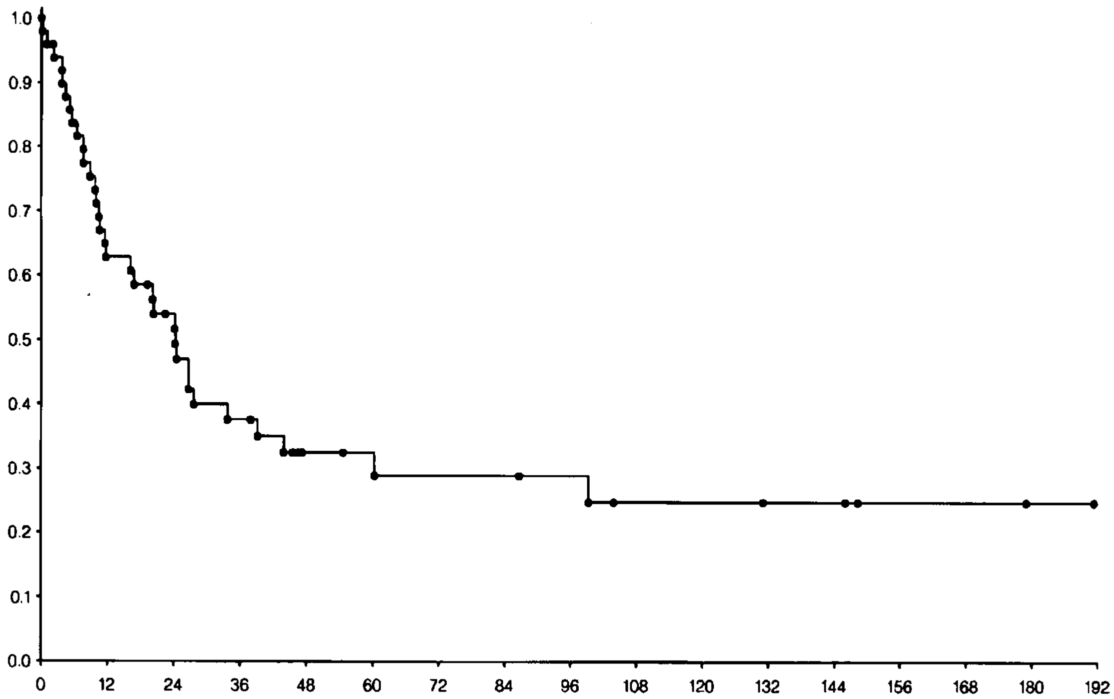

**Fig. 1.** *Kaplan–Meier 5-year overall survival (months).*

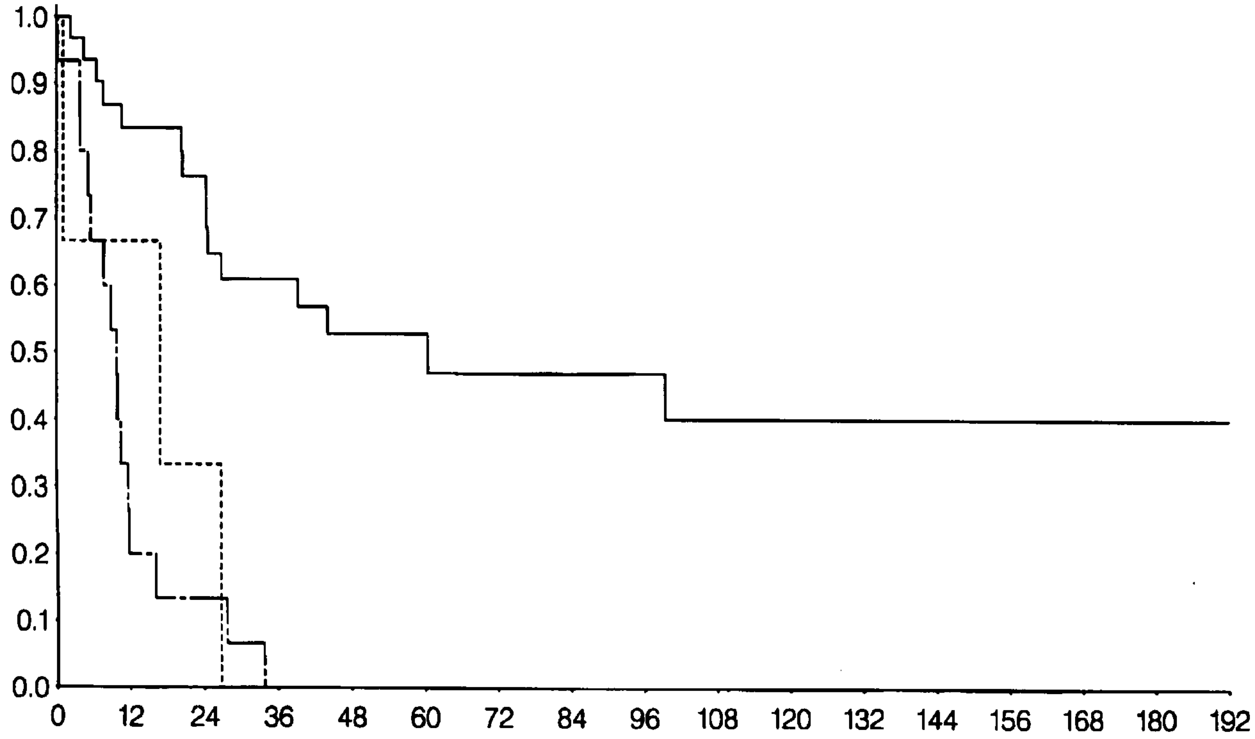

**Fig. 2.** *Kaplan–Meier 5-year survival (months) stratified by presentation status: dot-dashed line, metastatic presentation; dotted line, recurrent presentation; solid line, primary presentation.*

re-resection specimen.[15] Unfortunately, location alone may limit the extent of the initial resection for patients with angiosarcoma and re-resection is similarly not feasible.

In this analysis, there was no observed significant outcome advantage based on tumor site. In the group of patients with HNS disease ($n$=16), 6/16 (35%) patients developed local recurrence, two developed metastases and 11 (65%) died of their disease. In a previous report of patients ($n$=18) with angiosarcoma of the HNS,[14] only 50% of the patients had resectable disease at presentation and, following resection, approximately 30% of these patients were found to have positive microscopic margins.[14] The reported overall 5-year survival in that series of patients was 33% and the factor predictive of a negative survival was limited to size >10 cm.

The 2- and 5-year overall survival for patients with angiosarcoma in this report ($n$=50) was 50 and 30%, respectively. Angiosarcoma has been suggested to have the poorest prognosis among the soft tissue sarcomas,[5,13,16] and our survival data supports this observation. However, prolonged survival may be achieved in selected patients; among the nine patients who survived at least 5 years, five survived longer than 10 years (131, 146, 148, 178, and 191 months). The patients observed to have long-term survival all presented with surgically resectable primary tumors of the extremities, breast and head and neck. There were no long-term survivors in the group of patients presenting with primary thoracic, visceral or retroperitoneal disease.

Outcome for patients with STS may reflect differences in surgical resection of disease, tumor grade, the lack of efficacious adjuvant therapy or the inherent biologic properties of the tumor,[1,11,12,17] as has been proposed in previous reports. The observed patterns of disease progression in this series suggest a tendency for metastasis rather than local recurrence for patients with angiosarcoma; however, factors influencing local recurrence and metastatic patterns are precluded by the modest cohort in this analysis.

Additionally, the strict pathologic review of tissue specimens prior to inclusion into this analysis may have also prevented the confirmation of previously reported prognostic factors for patients with angiosarcoma.

### Conclusions

Angiosarcoma is an uncommon tumor that is biologically aggressive, with a high metastatic potential and subsequent mortality. Patients presenting with primary angiosarcoma have a significant survival advantage compared with patients with recurrent or metastatic disease. Patients with primary angiosarcoma should undergo surgical resection for a potential survival advantage. Long-term survival for patients with primary angiosarcoma undergoing resection is possible, as evidenced by the results of this analysis. Patients with recurrent or metastatic angiosarcoma should be considered to be at high-risk for progression of the disease and we would recommend enrollment into investigational trials to define the role of postoperative adjuvant therapies and potentially improve survival.

### Acknowledgment

Supported by NIH Grant CA-47179.

### References

1 Enzinger FM, Weiss SW. *Soft Tissue Tumors*. New York: Mosby, 1995.
2 Weiss SW, Enzinger FM. Epithelioid hemangioendothelioma. A vascular tumor often mistaken for a carcinoma. *Cancer* 1982; 50:971–81.
3 Fletcher CDM. Vascular tumors. An update with emphasis on the diagnosis of angiosarcoma and borderline vascular neoplasms. In: Weiss SW, Brooks JSJ, eds. *Soft Tissue Tumors*, Baltimore: Williams and Wilkins, 1996.
4 Rosenberg SA, Yang JC, Glatstein EJ, Antman KH. *Cancer. Principles and Practice of Oncology*. Philadelphia: J.B. Lippincott, 1993.
5 Maddox JC, Evans HL. Angiosarcoma of skin and soft tissue: a study of forty-four cases. *Cancer* 1981; 1907–21.
6 Taghian A, de Vathaire F, Terrier P, *et al*. Long-term risk of sarcoma following radiation treatment for breast cancer. *Int J Radiat Oncol Biol Phys* 1991; 21:361–7.
7 Lewis JJ, Brennan MF. Soft tissue sarcomas. *Curr Probl Surg* 1996; 33:817–72.
8 Donnell RM, Rosen PP, Lieberman PH, *et al*. Angiosarcoma and other vascular tumors of the breast. Pathologic analysis as a guide to prognosis. *Am J Surg Pathol* 1981; 5:629–42.
9 Kaplan EL, Meier P. Nonparametric estimation from incomplete observations. *J Am Statist Assoc* 1958; 53:457–62.
10 Naka N, Ohsawam, Tomita Y, Kanno H, Uchida A, Aozasa K. Angiosarcoma in Japan. A review of 99 cases. *Cancer* 1995; 75:989–96.
11 Brennan MF. The enigma of local recurrence. The Society of Surgical Oncology. *Ann Surg Oncol* 1997; 4:1–12.
12 Lewis JJ, Leung D, Heslin M, Woodruff JM, Brennan MF. Association of local recurrence with subsequent survival in extremity soft tissue sarcoma. *J Clin Oncol* 1997; 15:646–52.
13 Karpeh MS, Caldwell C, Gaynor JJ, *et al*. Vascular soft-tissue sarcomas. An analysis of tumor-related mortality. *Arch Surg* 1991; 126:1474–81.
14 Lydiatt WM, Shaha AR, Shah JP. Angiosarcoma of the head and neck. *Am J Surg* 1994; 168:451–4.
15 Giuliano AE, Eilber FR. The rationale for planned reoperation after unplanned total excision of soft-tissue sarcomas. *J Clin Oncol* 1985; 3:1344–8.
16 Naka N, Ohsawa M, Tomita Y, Uchida A. Prognostic factors in Angiosarcoma: a multivariate analysis of 55 cases. *J Surg Oncol* 1996; 61:170–6.
17 Brennan MF, Capser ES, Harrison LB, Shiu MH, Gaynor J, Hajdu SI. The role of multimodality therapy in soft tissue sarcoma. *Ann Surg* 1991; 214:328–36.
